# Supplementary material for: RAISING is a high-performance method for identifying random transgene integration sites
Source: Commun Biol. 2022 Jun 2;5:535. doi: 10.1038/s42003-022-03467-w (PMC9163355; doi:10.1038/s42003-022-03467-w)
Supplement: Supplementary file 1 — Supplementary Information [file 42003_2022_3467_MOESM1_ESM.pdf]

Supplementary Information

RAISING is a high-performance method for identifying random transgene integration sites

Yusaku Wada[1,†], Tomoo Sato[2,3,†], Hiroo Hasegawa[4,5,†], Takahiro Matsudaira[1,†], Naganori Nao[6,7], Ariella L. G. Coler-Reilly[2,8], Tomohiko Tasaka[9], Shunsuke Yamauchi[4], Tomohiro Okagawa[10], Haruka Momose[11], Michikazu Tanio[11], Madoka Kuramitsu[11], Daisuke Sasaki[4], Nariyoshi Matsumoto[4], Naoko Yagishita[2], Junji Yamauchi[2], Natsumi Araya[2], Kenichiro Tanabe[12], Makoto Yamagishi[13], Makoto Nakashima[13], Shingo Nakahata[14], Hidekatsu Iha[15], Masao Ogata[16], Masamichi Muramatsu[17], Yoshitaka Imaizumi[18], Kaoru Uchimaru[13], Yasushi Miyazaki[18,19], Satoru Konnai[10,20], Katsunori Yanagihara[4,5], Kazuhiro Morishita[14], Toshiki Watanabe[21], Yoshihisa Yamano[2,3,*], Masumichi Saito[17,22,*]

[1]Biotechnological Research Support Division, FASMAC Co., Ltd, 243-0021 Kanagawa, Japan

[2]Department of Rare Diseases Research, Institute of Medical Science, St. Marianna University School of Medicine, 216-8512 Kanagawa, Japan

[3]Division of Neurology, Department of Internal Medicine, St. Marianna University School of Medicine, 216-8511 Kanagawa, Japan

[4]Department of Laboratory Medicine, Nagasaki University Hospital, 852-8501 Nagasaki, Japan

[5]Department of Laboratory Medicine, Nagasaki University Graduate School of Biomedical Sciences, 852-8501 Nagasaki, Japan

[6]Division of International Research Promotion, International Institute for Zoonosis Control, Hokkaido University, Sapporo 001-0020, Japan

[7]One Health Research Center, Hokkaido University, Sapporo 060-0818, Japan

[8]Department of Internal Medicine, Division of Bone and Mineral Diseases, Washington University School of Medicine, St. Louis, MO, 63110 USA

[9]Affinity Science Corporation, 141–0031 Tokyo, Japan

[10]Department of Advanced Pharmaceutics, Faculty of Veterinary Medicine, Hokkaido University, 060-0818 Hokkaido, Japan

[11]Department of Safety Research on Blood and Biological Products, National Institute of

Infectious Diseases, 208-0011 Tokyo, Japan
[12]Pathophysiology and Bioregulation, St. Marianna University Graduate School of Medicine, 216-8511 Kanagawa, Japan
[13]Department of Computational Biology and Medical Sciences, Graduate School of Frontier Sciences, The University of Tokyo, 108-8639 Tokyo, Japan
[14]Division of Tumor and Cellular Biochemistry, Department of Medical Sciences, University of Miyazaki, 889-1692 Miyazaki, Japan.
[15]Department of Microbiology, Faculty of Medicine, Oita University, 879-5593 Oita, Japan
[16]Department of Hematology, Oita University Hospital, 879-5593 Oita, Japan
[17]Department of Virology II, National Institute of Infectious Diseases, 162-8640 Tokyo, Japan
[18]Department of Hematology, Nagasaki University Hospital, 852-8501 Nagasaki, Japan
[19]Atomic Bomb Disease and Hibakusha Medicine Unit, Atomic Bomb Disease Institute, Nagasaki University, 852-8102 Nagasaki, Japan
[20]Department of Disease Control, Faculty of Veterinary Medicine, Hokkaido University, Sapporo, 060-0818 Hokkaido, Japan
[21]Department of Practical Management of Medical Information, St. Marianna University Graduate School of Medicine, 216-8511 Kanagawa, Japan
[22]Center for Emergency Preparedness and Response, National Institute of Infectious Diseases, 162-8640 Tokyo, Japan
[†]These authors contributed equally: Yusaku Wada, Tomoo Sato, Hiroo Hasegawa, and Takahiro Matsudaira
[*]These authors jointly supervised this work: Yoshihisa Yamano and Masumichi Saito
Correspondence: Masumichi Saito (saitomas@niid.go.jp and saitomas@nih.go.jp)

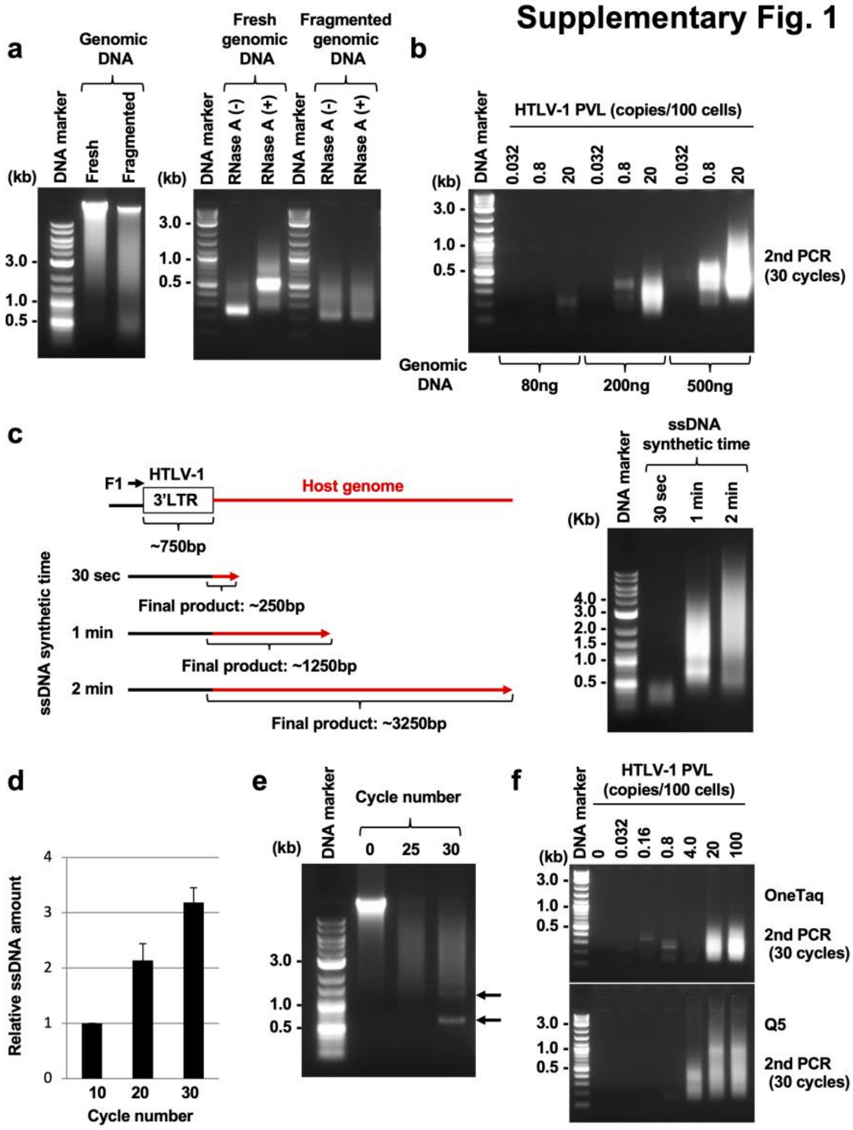

Supplementary Fig. 1. Examination of step 1 in Rapid Amplification of Integration Sites without Interference by Genomic DNA contamination (RAISING). **a.** Two genomic DNA samples, freshly isolated and fragmented genomic DNA, were prepared to examine the effect of DNA quality on RAISING (Left panel, electrophoresis on 0.6% agarose gel). The effect of RNase A-treatment on RAISING was also examined using the two genomic DNAs (Right panel). RAISING products were visualized using electrophoresis on 2% agarose gel. **b.** RAISING was performed using a different amount of genomic DNA in Fig. 2a. RAISING products were visualized using electrophoresis on 2% agarose gels. **c.** Examination of extension time for single-stranded DNA (ssDNA) synthesis in step 1 of RAISING. Schematic representation of the estimated final PCR product sizes according to the ssDNA synthetic time using KOD-Plus-Neo (Left panel). RAISING was performed using TL-Om1 (HTLV-1-infected cell line) genomic DNA, and the products were visualized using electrophoresis on 2% agarose gels (Right panel). **d.** In step 1 of RAISING, the ssDNA synthesis was performed with the indicated cycle numbers using TL-Om1 genomic DNA. The relative amount of ssDNA was measured using qPCR with primers for the HTLV-1 3′ long terminal repeat. Data are shown as mean ± SD of three independent qPCRs. **e.** In step 1 of RAISING, the ssDNA synthesis was examined with the indicated cycle numbers using an HTLV-1-specific F1 primer and TL-Om1 genomic DNA. The arrows indicate non-specific amplifications. Genomic DNA and non-specific products were visualized using electrophoresis on 0.6% agarose gels. **f.** Step 1 of RAISING was performed using the same genomic DNAs as shown in Fig. 2a and two PCR enzymes: OneTaq and Q5. RAISING products were visualized using electrophoresis on 2% agarose gels.

Supplementary Fig. 2

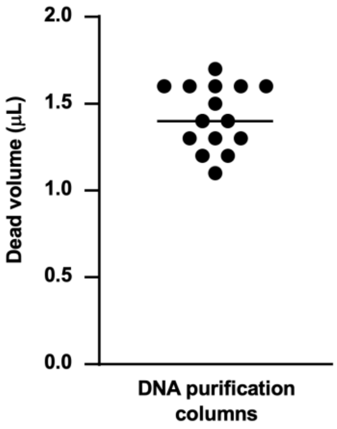

Supplementary Fig. 2. Examination of step 2 in Rapid Amplification of Integration Sites without Interference by Genomic DNA contamination (RAISING). The Dead volume of DNA purification columns in Monarch PCR & DNA cleanup kit was examined in step 2 of RAISING. The average dead volume was obtained using 15 individual columns.

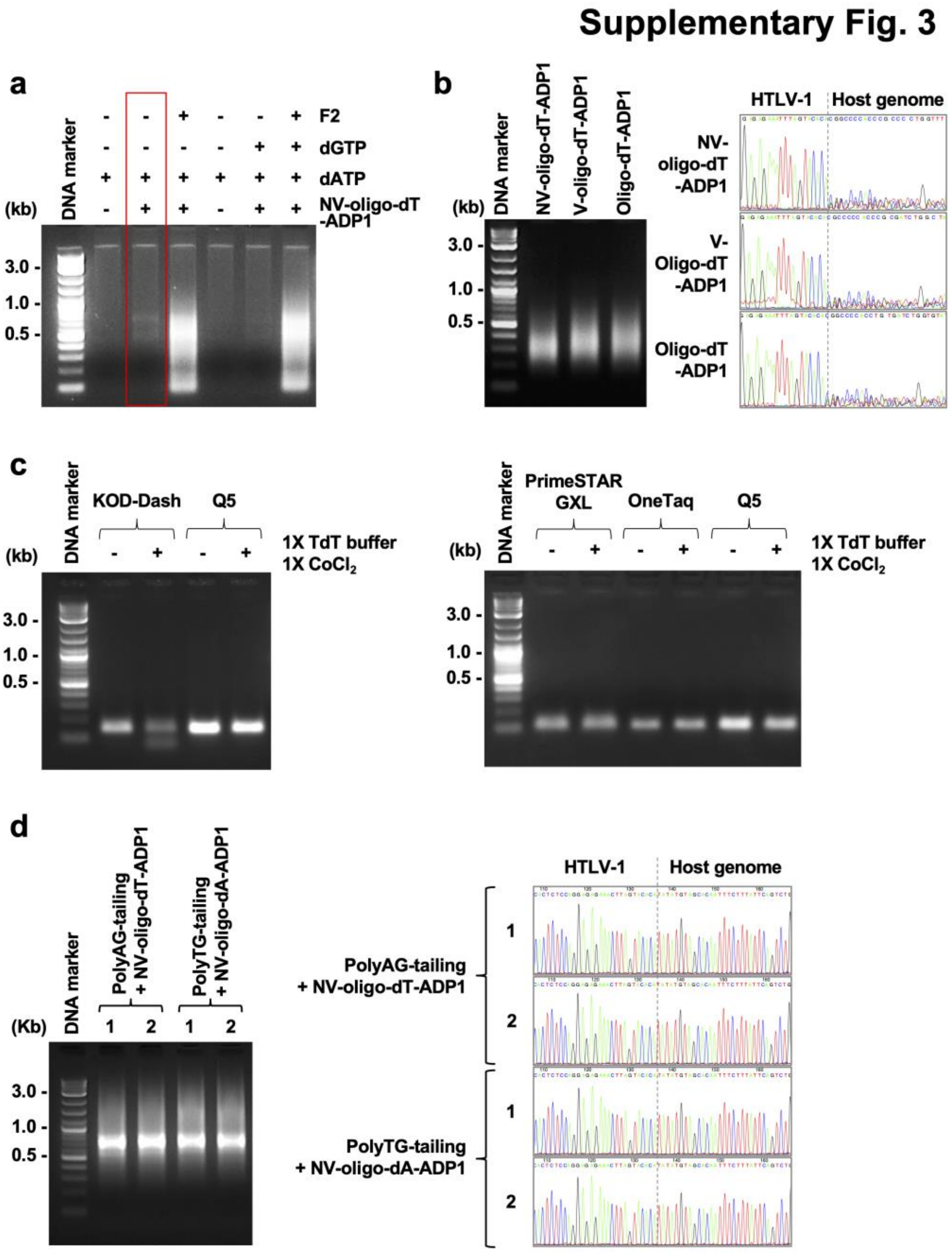

Supplementary Fig. 3. Examination of steps 3–5 in Rapid Amplification of Integration Sites without Interference by Genomic DNA contamination (RAISING). **a.** The effect of polyAG-tailing was examined in steps 3–5 of RAISING. The red box shows the lane, including non-specific amplifications. **b.** Oligo-dT-ADP1 primers, NV-oligo-dT-ADP1 that includes VN (V = A or G or C, N = A or G or C or T), V-oligo-dT-ADP1, and oligo-dT-ADP1 that included or did not include V at each 3′ -end, respectively, were examined in step 4 and 5 of RAISING using genomic DNA of SLB-1 (HTLV-1-infected cell line) harboring multiple HTLV-1 integration sites. Visualization of PCR products (Left panel). Sanger sequencing analysis of HTLV-1 integration sites (Right panel). **c.** Four PCR enzymes (KOD-Dash, PrimeSTAR-GXL, OneTaq, and Q5) were examined in steps 4 and 5 of RAISING. TdT, terminal transferase. **d**. Comparison of PolyAG-tailing with NV-oligo-dT-ADP1 and PolyTG-tailing with NV-oligo-dA-ADP1 in steps 3–5 of RAISING. RAISING was performed using TL-Om1 (HTLV-1-infected cell line) genomic DNA. Data from two independent experiments (1 and 2) are shown. Visualization of PCR products (Left panel). Sanger sequencing analysis of HTLV-1 integration sites (Right panel). All PCR products were visualized using electrophoresis on 2% agarose gels.

Supplementary Fig. 4

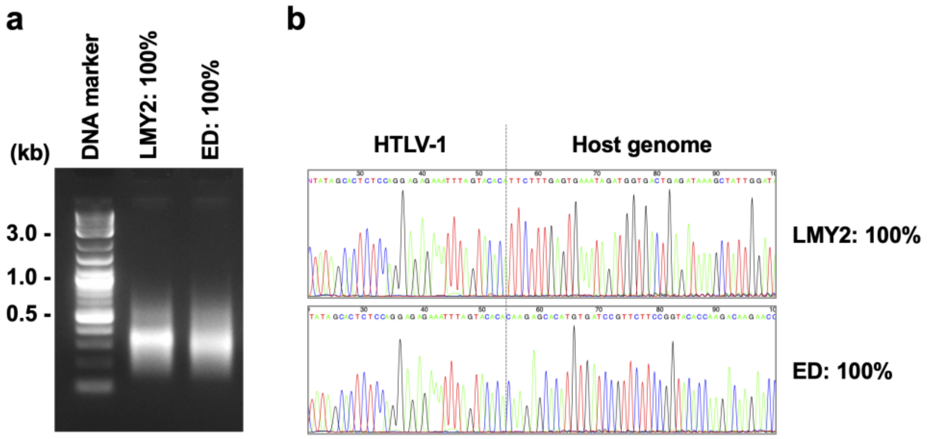

Supplementary Fig. 4. Amplification of HTLV-1 integration sites in HTLV-1-infected cell lines with Rapid Amplification of Integration Sites without Interference by Genomic DNA contamination (RAISING). **a**. Genomic DNAs of LMY2 and ED harboring a single copy of HTLV-1 were used for RAISING. PCR products were visualized using electrophoresis on 2% agarose gels. **b**. HTLV-1 integration sites in LMY2 and ED were analyzed using Sanger sequencing.

Supplementary Fig. 5

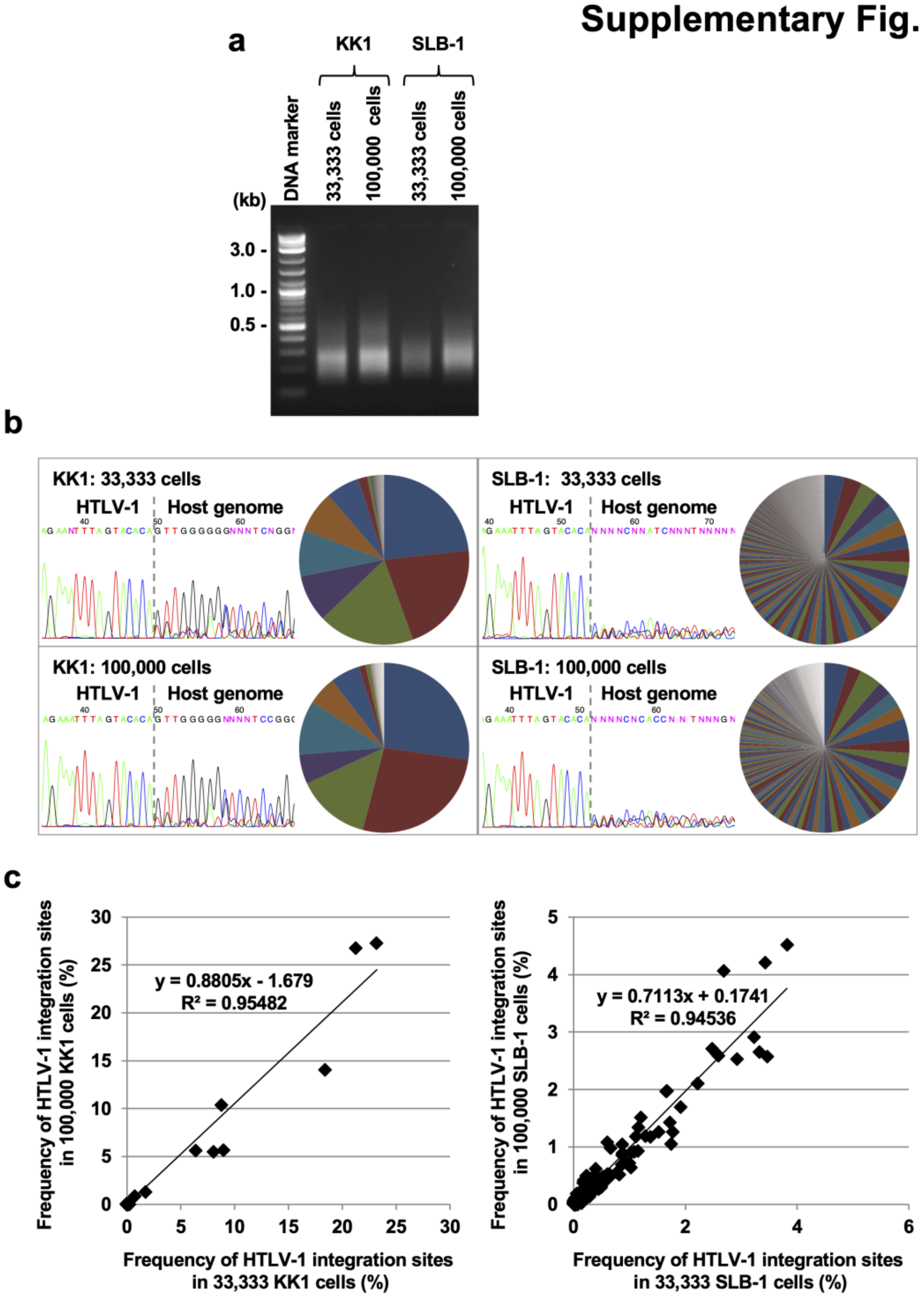

Supplementary Fig. 5. Accuracy and utility of Rapid Amplification of Integration Sites without Interference by Genomic DNA contamination (RAISING). **a.** Genomic DNAs of KK1 and SLB-1 (HTLV-1 infected cell lines) harboring multiple HTLV-1 integration sites were extracted with a cell-direct method at the indicated cell number, and the genomic DNAs ware sequentially used for RAISING. PCR products were visualized using electrophoresis on 2% agarose gels. **b.** HTLV-1 integration sites in KK1 and SLB-1 were analyzed using Sanger and high throughput sequencing (HTS). **c.** HTS analysis was conducted to evaluate the accuracy of RAISING in **a** and **b**. The correlation coefficient was determined by calculating the frequency of each HTLV-1 integration site in each HTS dataset.

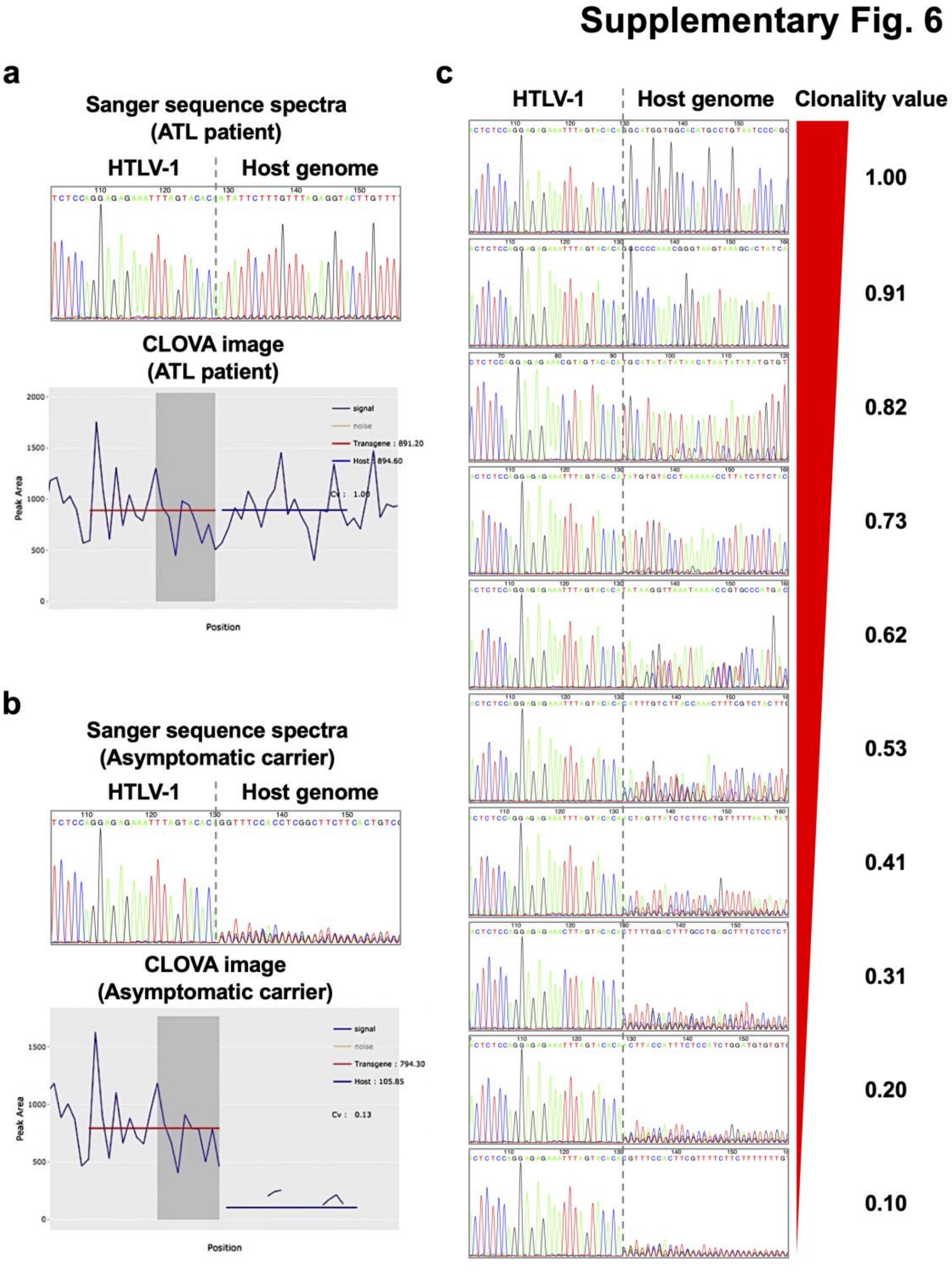

Supplementary Fig. 6. Characterization of CLOVA, a software developed to analyze the clonality value of HTLV-1-infected cells. **a.** A representative result of Sanger sequence spectra of HTLV-1-infected cells in an adult T-cell leukemia/lymphoma (ATL) patient and the CLOVA image. **b.** A representative result of HTLV-1-infected cells in an asymptomatic carrier and the CLOVA image. **c**. Comparison of Sanger sequence spectra patterns and clonality values analyzed using CLOVA.

Supplementary Fig. 7

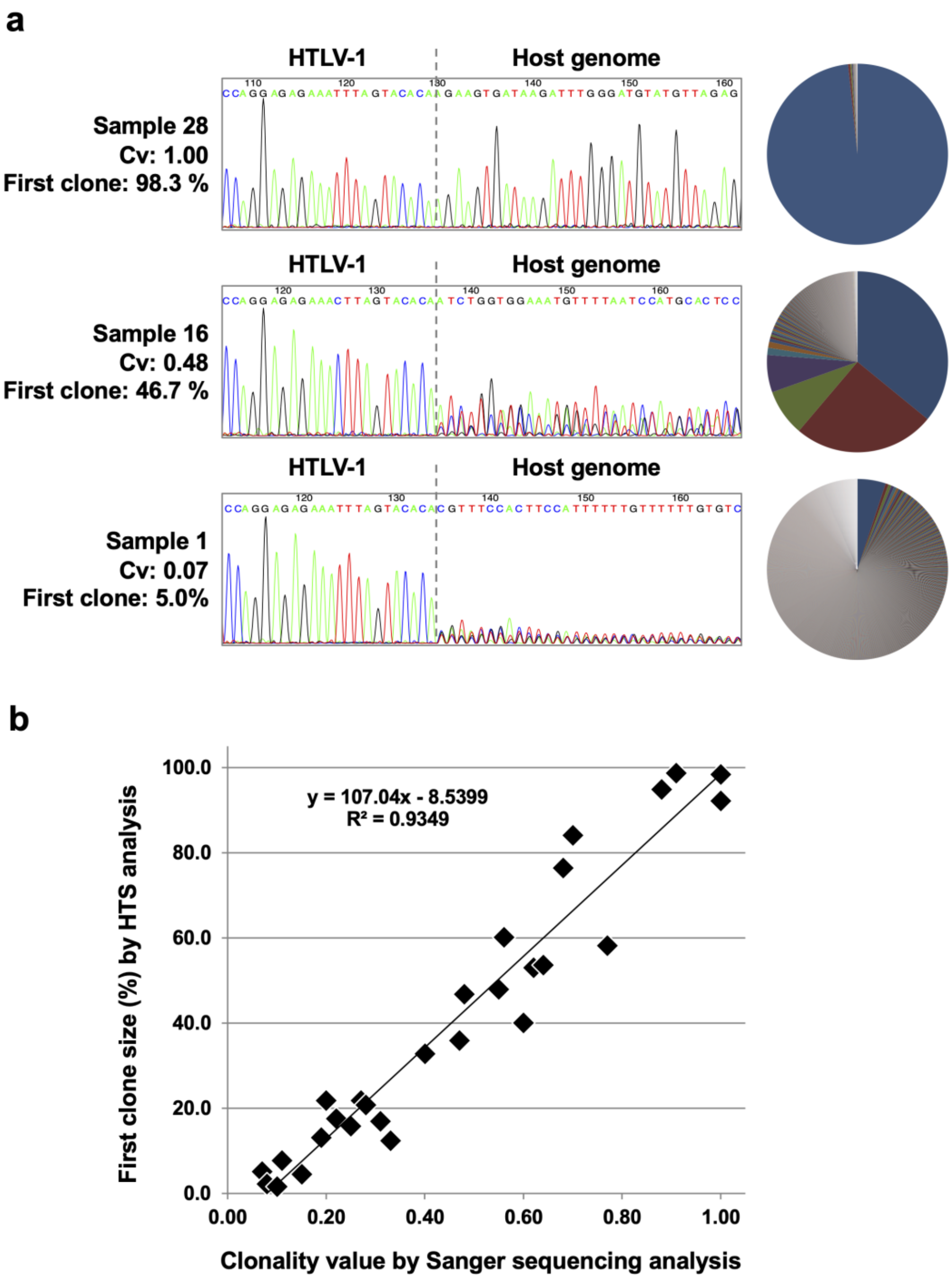

Supplementary Fig. 7. Comparison of clonality values and the first clone sizes in HTLV-1-infected samples. Clonality values and the clone sizes in HTLV-1-infected samples (n = 28) were analyzed using Rapid Amplification of Integration Sites without Interference by Genomic DNA contamination (RAISING) with Sanger sequencing and high throughput sequencing, respectively. **a.** Samples 1, 16, and 28 are shown as examples for analyzing clonality values and the first clone sizes. Pie charts indicate the variety and size of HTLV-1-infected clones. The first clone is shown with dark blue color in each pie chart. **b.** Positive correlation of clonality values and the first clone sizes. The correlation coefficient was determined using Microsoft Excel for Mac 2011 (ver.14.7.1) (95% confidence intervals of slope and intercept are 95.65 to 118.42 and –14.67 to –2.41, respectively).

Supplementary Fig. 8

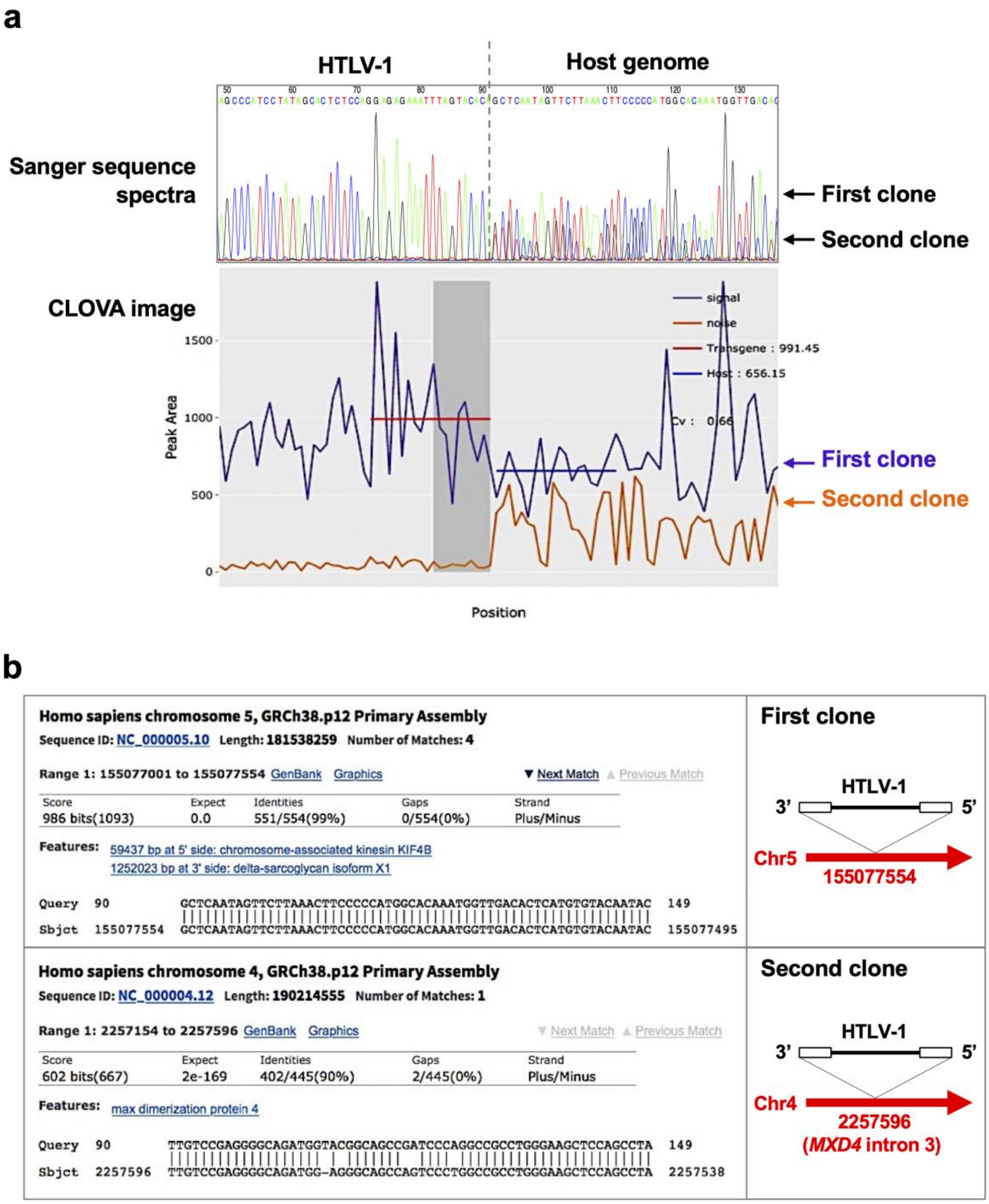

Supplementary Fig. 8. Identification of HTLV-1 integration sites in the first and second clones using CLOVA. **a.** Example of Sanger sequence spectra showing the presence of the first and second clones in an adult T-cell leukemia/lymphoma (ATL) patient. An image of the first and second clones in CLOVA is shown. **b**. Results of a BLAST homology search for the first and second clones in **a**. The red arrows and scheme of HTLV-1 indicate the direction of nucleotide number on chromosomes and HTLV-1 provirus, respectively. The numbers under the red arrows show the chromosomal nucleotide number of each HTLV-1 integration site.

Supplementary Fig. 9

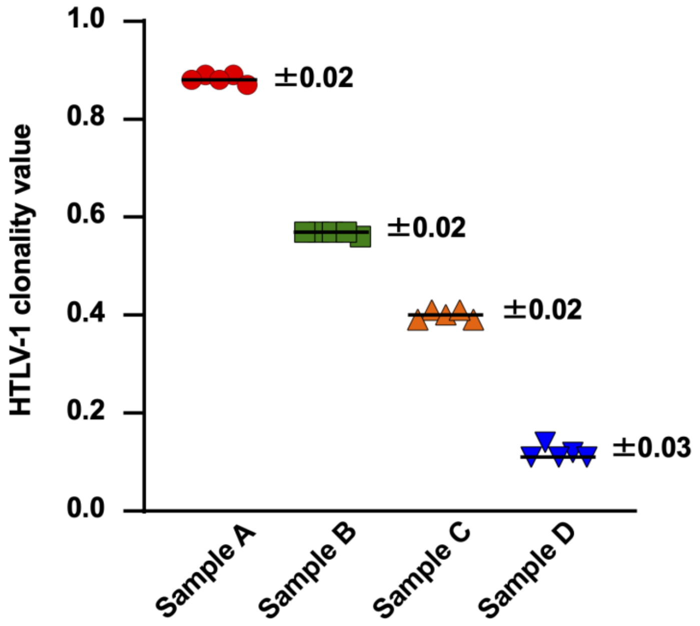

Supplementary Fig. 9. Accuracy and consistency of Rapid Amplification of Integration Sites without Interference by Genomic DNA contamination (RAISING)-CLOVA. Variation of HTLV-1 clonality values among five Sanger sequence reactions. RAISING-CLOVA was performed with four samples harboring different HTLV-1 clonality values.

Supplementary Fig. 10

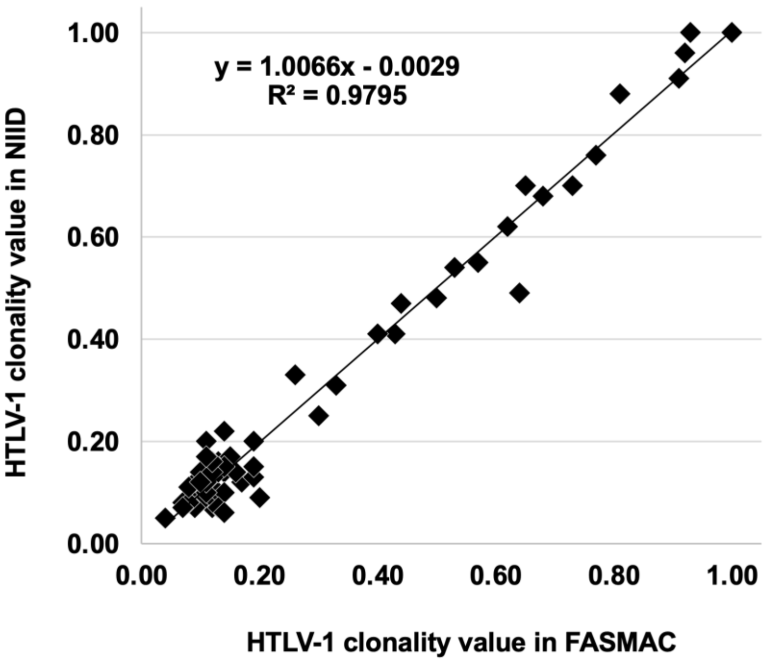

Supplementary Fig. 10. Comparison of HTLV-1 clonality values in two different laboratories. Rapid Amplification of Integration Sites without Interference by Genomic DNA contamination (RAISING) was independently performed at the National Institute of Infectious Diseases (NIID), and FASMAC, Co., Ltd. (FASMAC) using the same HTLV-1-infected specimens (n = 62), and HTLV-1 clonality values were determined using CLOVA. Comparison of the clonality values and correlation coefficient are shown (95% confidence intervals of slope and intercept are 0.97 to 1.04 and –0.02 to 0.01, respectively).

Supplementary Fig. 11

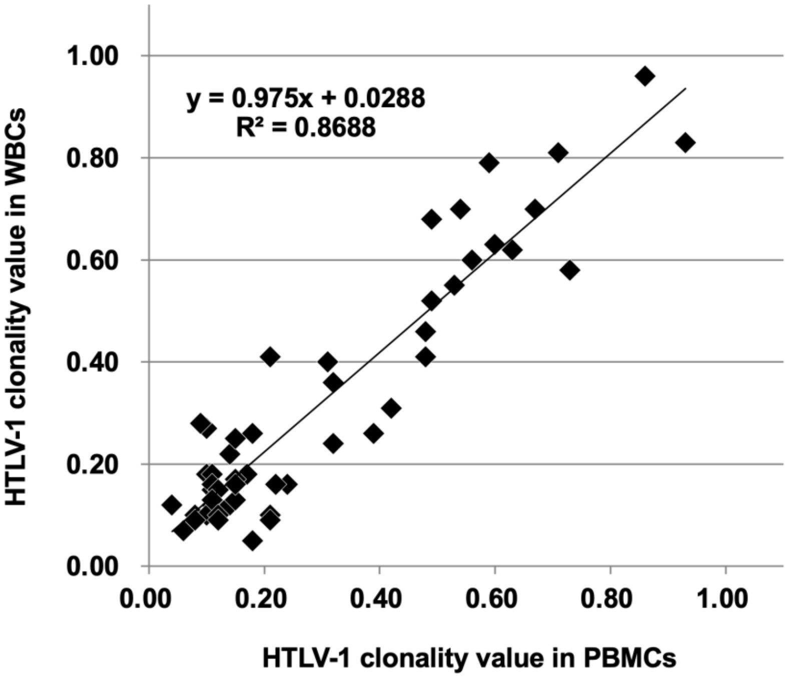

Supplementary Fig. 11. Comparison of HTLV-1 clonality values between whole blood cells (WBCs) and peripheral blood mononuclear cells (PBMCs). Rapid Amplification of Integration Sites without Interference by Genomic DNA contamination (RAISING)-CLOVA was performed using samples of WBCs and PBMCs from the same patients (n = 53). Comparison of the clonality values and correlation coefficient are shown (95% confidence intervals of slope and intercept are 0.86 to 1.08 and –0.01 to 0.07, respectively).

Supplementary Fig. 12

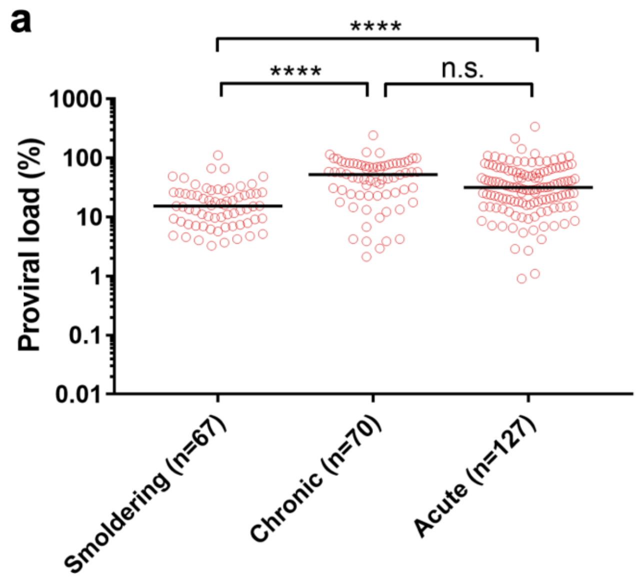

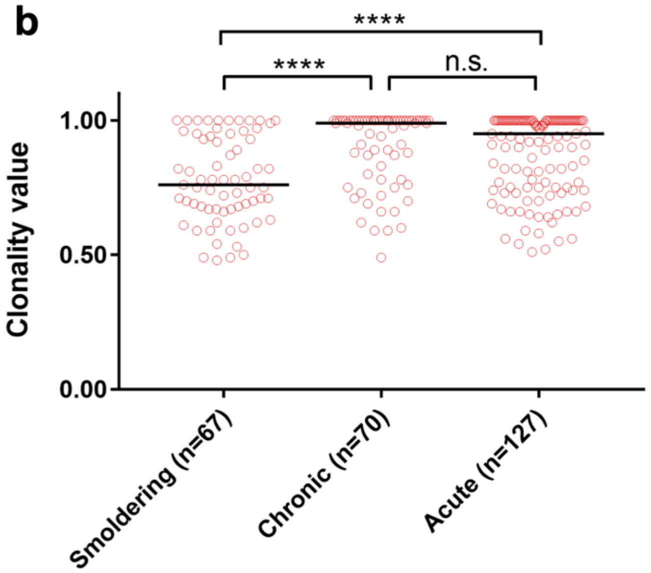

Supplementary Fig. 12. Comparison of HTLV-1 proviral loads and clonality values among ATL subtypes. HTLV-1 proviral load (**a**) and clonality value (**b**) derived from peripheral blood of ATL patients (n = 264) were compared among ATL subtypes. Horizontal bars indicate the median values. Statistical analysis was performed using the Kruskal–Wallis test followed by Dunn's post-hoc test: ****p < 0.0001, n.s., not significant.

Supplementary Fig. 13

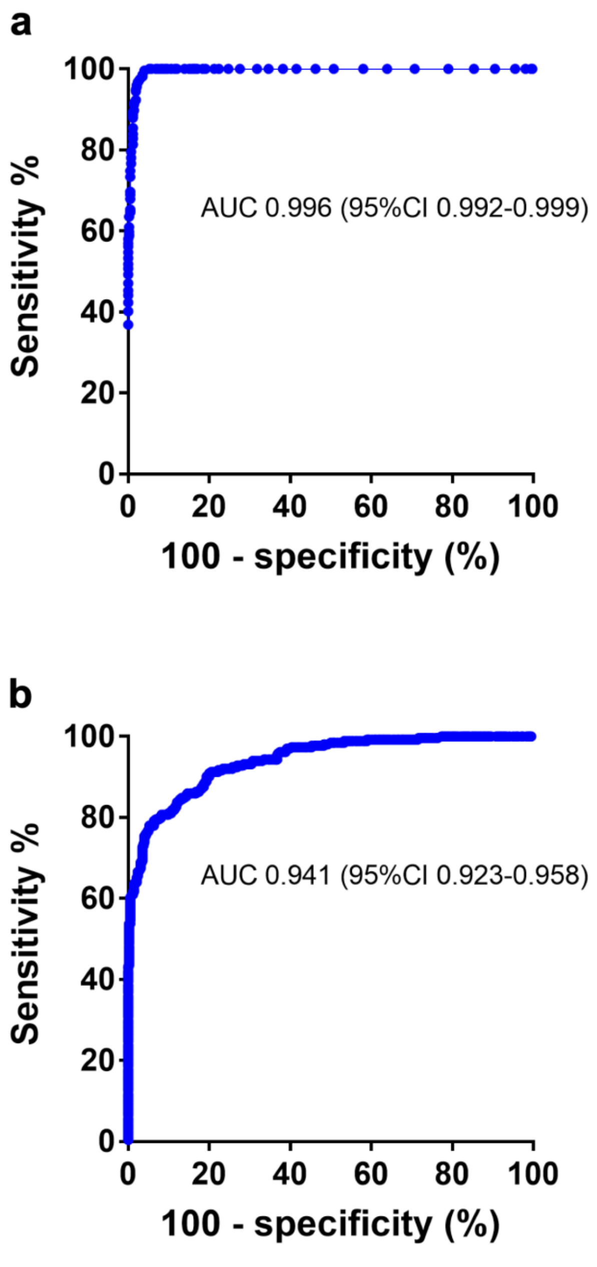

Supplementary Fig. 13. Receiver operating characteristic (ROC) curve analysis with clonality values and HTLV-1 proviral loads among asymptomatic carriers (AC) and HTLV-1-associated myelopathy/tropical spastic paraparesis (HAM/TSP) and ATL patients. **a.** Ability of HTLV-1 clonality value to discriminate ATL from both AC and HAM/TSP. **b.** Ability of HTLV-1 proviral load to discriminate ATL from both AC and HAM/TSP. AUC, area under the ROC curve; 95% CI, 95% confidence interval.

Supplementary Fig. 14

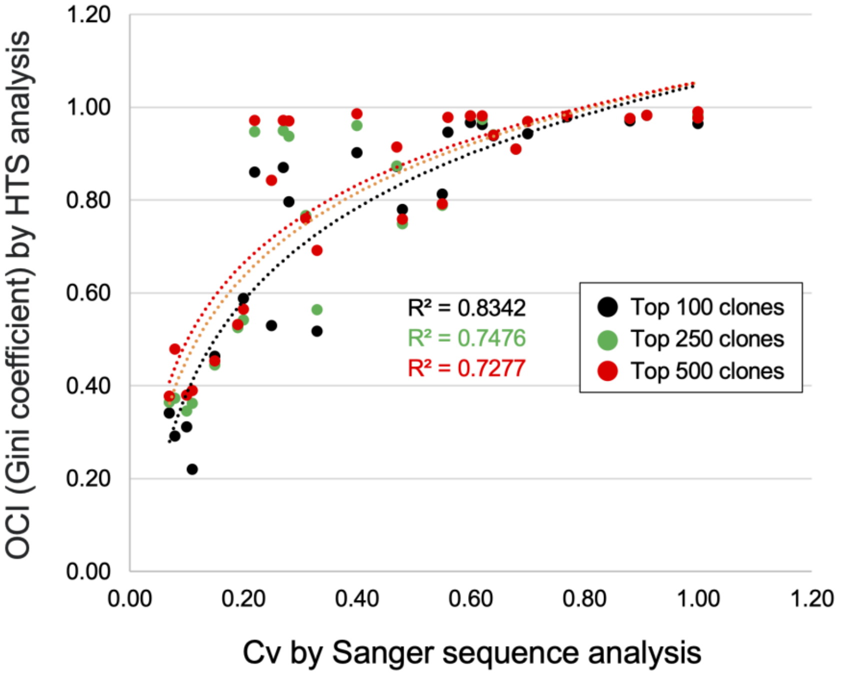

Supplementary Fig. 14. Correlation between clonality value (Cv) and oligoclonal index (OCI). The high throughput sequencing data in Supplementary Fig. 7 was used to calculate OCI. The correlation between Cv and OCI was analyzed with three different numbers of top clones. The correlation coefficient was determined using Microsoft Excel for Mac 2011 (ver.14.7.1)

Supplementary Fig. 15

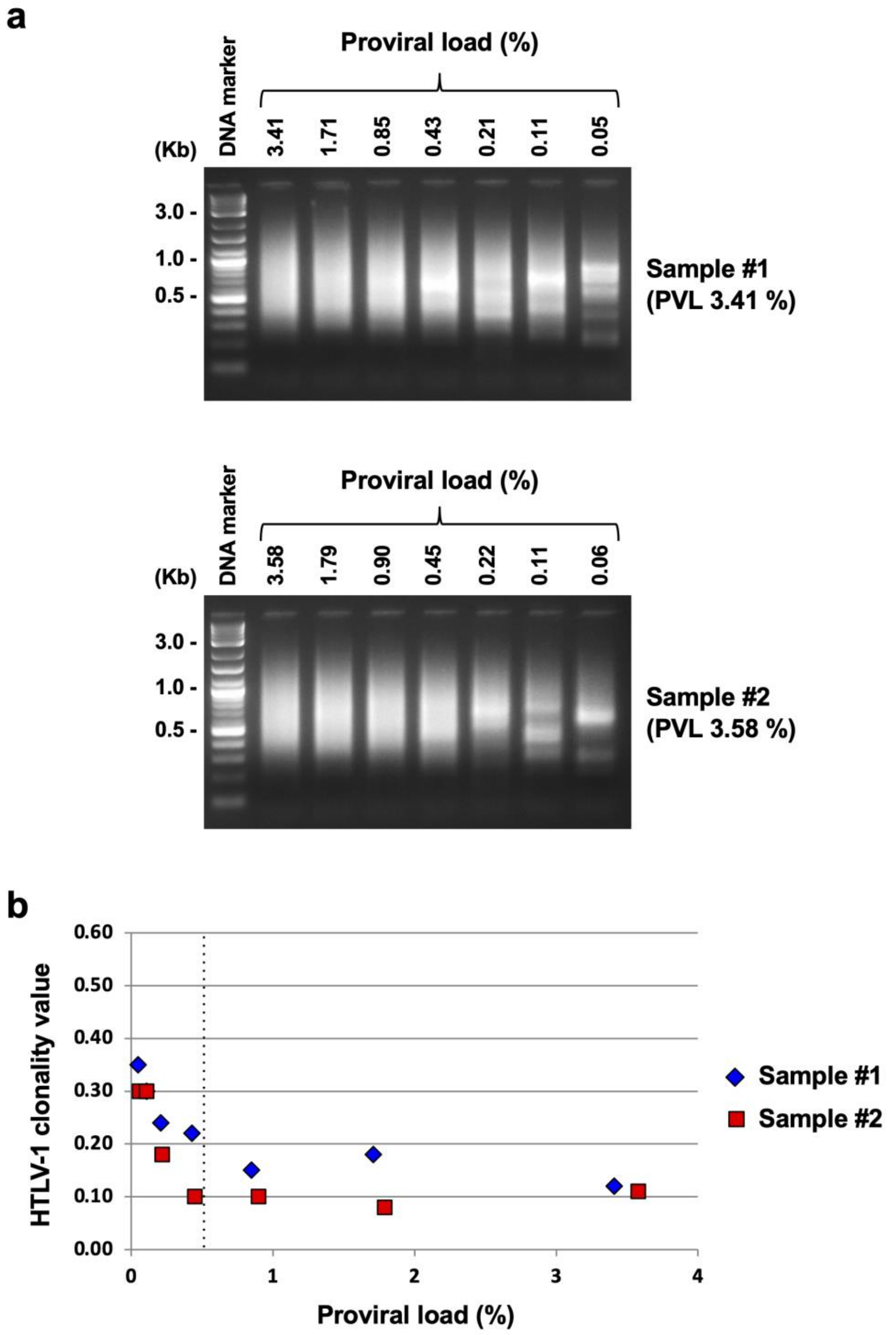

Supplementary Fig. 15. The minimum HTLV-1 proviral load (PVL) was examined to measure a reliable clonality value with Rapid Amplification of Integration Sites without Interference by Genomic DNA contamination (RAISING)-CLOVA. **a.** RAISING-CLOVA was performed using genomic DNA of two HTLV-1-infected specimens (Samples #1 and #2) serially diluted with genomic DNA of peripheral blood mononuclear cells (HTLV-1-negative cells). PCR products were visualized using electrophoresis on 2% agarose gels. **b.** Analysis of the clonality values and identification of the minimum PVL in **a**. The dotted line indicates the minimum PVL 0.5% for HTLV-1 clonality analysis with RAISING-CLOVA.

Supplementary Fig. 16

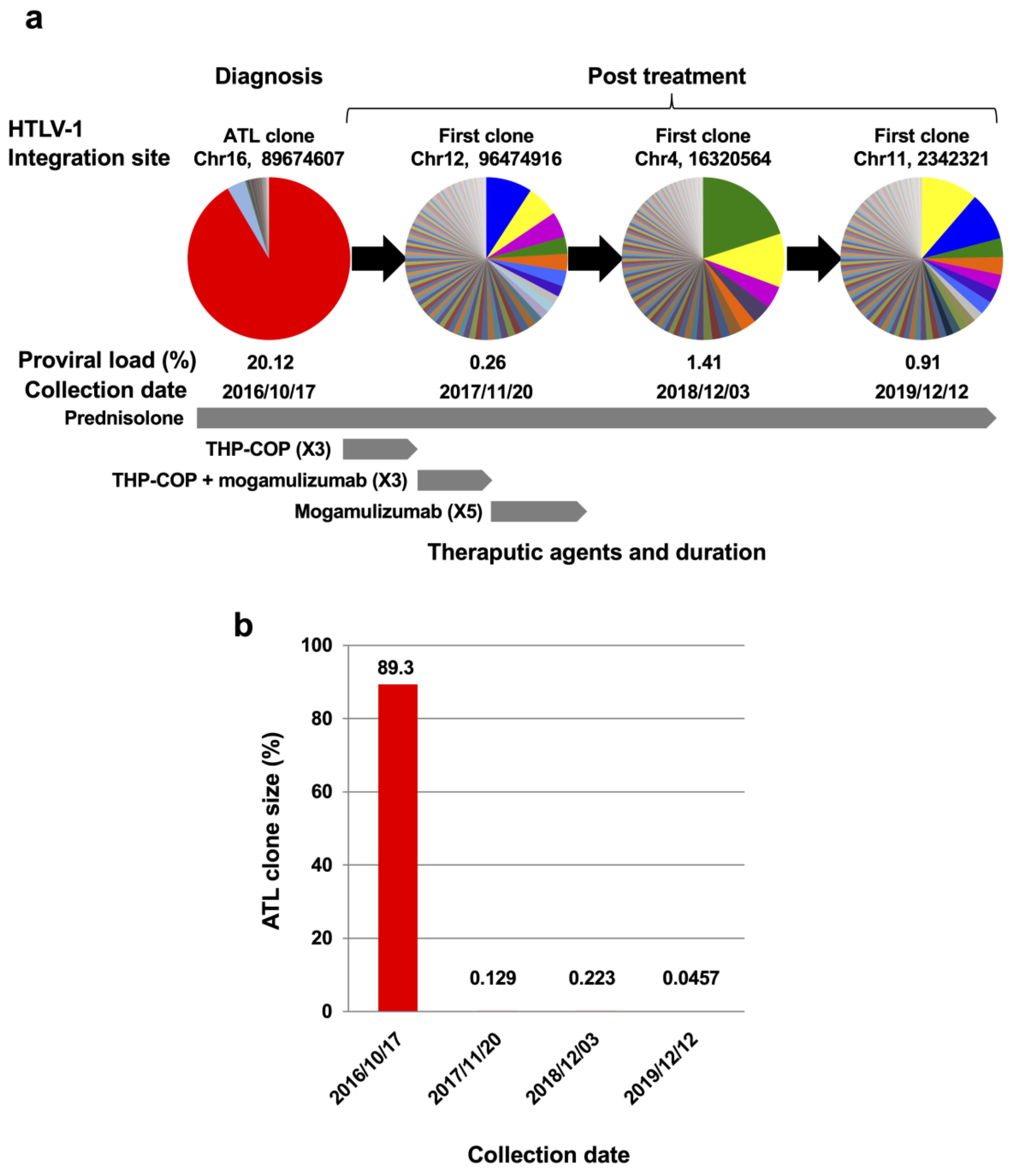

Supplementary Fig. 16. Clonal changes of HTLV-1-infected cells in an ATL patient (HAMATL001) before and after treatment. **a.** Rapid Amplification of Integration Sites without Interference by Genomic DNA contamination (RAISING) with high throughput sequencing was performed using four specimens collected on the indicated dates. The variety and size of HTLV-1-infected clones are shown in each pie chart with different colors. The same clone among the top 10 clones in different specimens is shown with the same color **b.** ATL clone size in **a**.

Supplementary Fig. 17

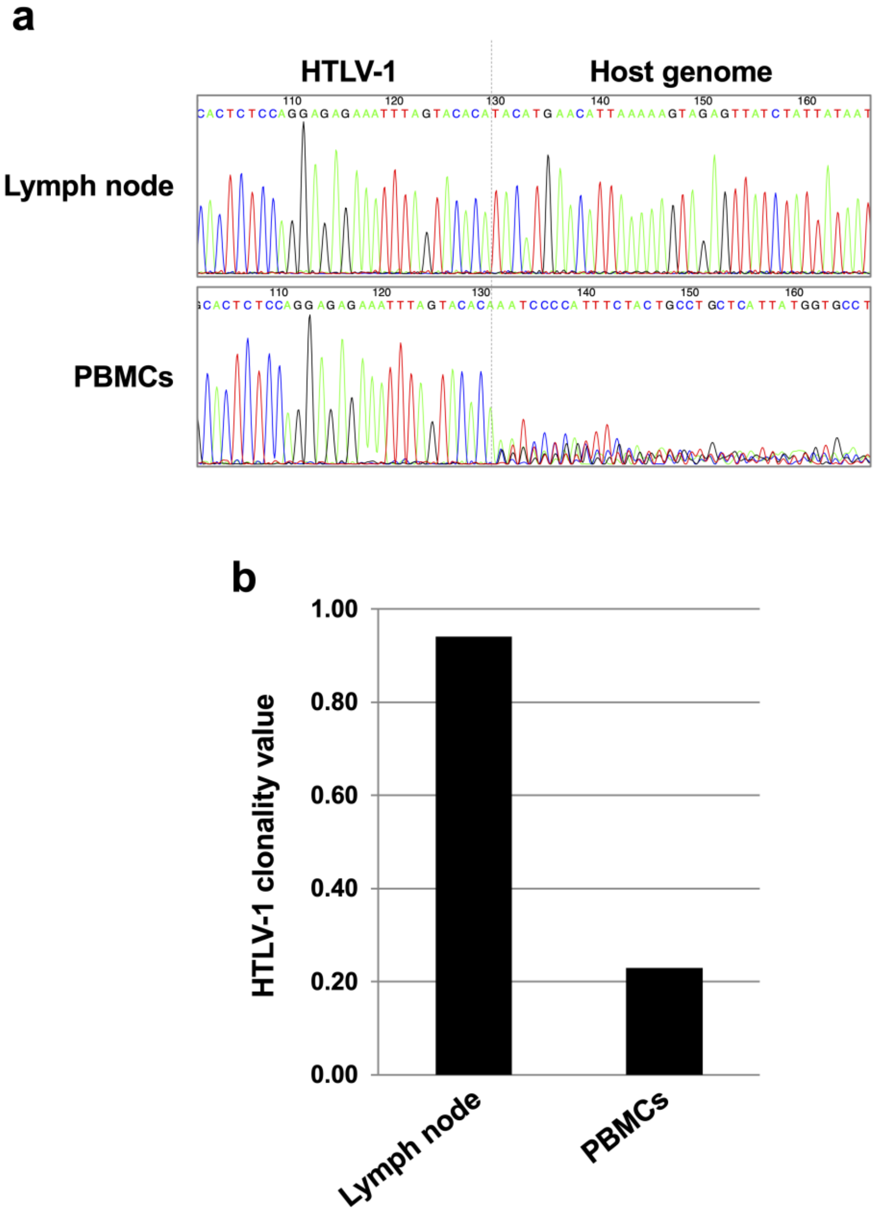

Supplementary Fig. 17. HTLV-1 clonality in a HAM/TSP patient (HAMATL004) who developed the lymphoma type of ATL. HTLV-1 clonality of HAMATL004 was analyzed with Rapid Amplification of Integration Sites without Interference by Genomic DNA contamination (RAISING)-CLOVA using specimens obtained from the lymph node and peripheral blood mononuclear cells (PBMCs). **a.** Sanger sequence spectra patterns of HTLV-1 clonality in the lymph node and PBMCs. **b.** The HTLV-1 clonality values of lymph node and PBMCs.

Supplementary Fig. 18

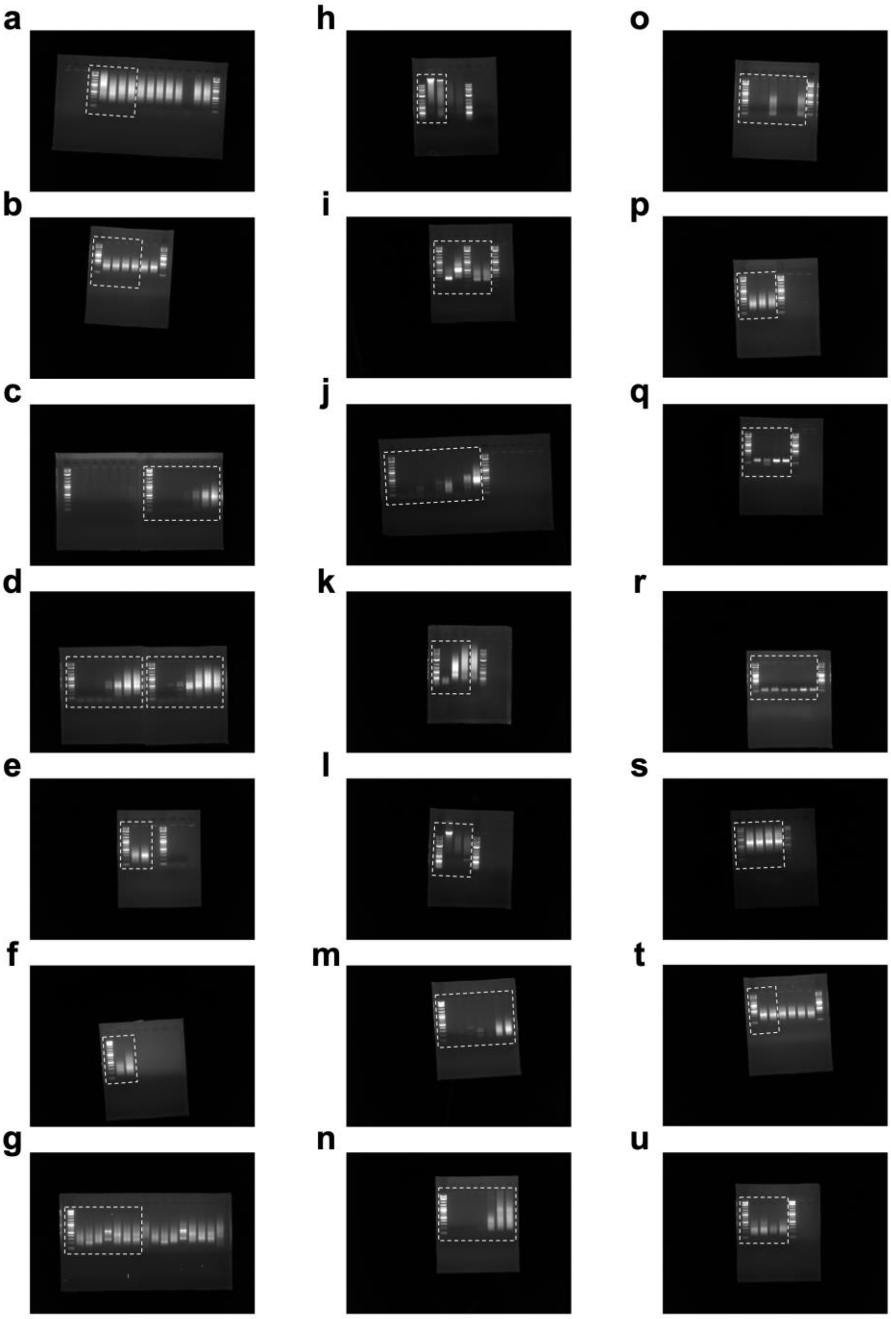

Supplementary Fig. 18. Uncropped gel images. **a.** Gel image for RAIS in Fig.1b. **b.** Gel image for RAISING in Fig. 1b. **c.** Gel image for 2nd PCR (26 cycles) in Fig. 2a. **d.** Gel image for 2nd PCR (29 and 32cycles) in Fig. 2a. **e.** Gel image in Fig. 2b. **f.** Gel image in Fig. 2c. **g.** Gel image in Fig. 2d. **h.** Gel image for genomic DNA in Supplementary Fig. 1a. **i.** Gel image for fresh and fragmented genomic DNAs in Supplementary Fig. 1a. **j.** Gel image in Supplementary Fig. 1b. **k.** Gel image in Supplementary Fig. 1c. **l.** Gel image in Supplementary Fig. 1e. **m.** Gel image for One Taq in Supplementary Fig. 1f. **n.** Gel image for Q5 in Supplementary Fig. 1f. **o.** Gel image in Supplementary Fig. 3a. **p.** Gel image in Supplementary Fig. 3b. **q.** Gel image for two PCR enzymes in Supplementary Fig. 3c. **r.** Gel image for three PCR enzymes in Supplementary Fig. 3c. **s.** Gel image in Supplementary Fig. 3d. **t.** Gel image in Supplementary Fig. 4a. **u.** Gel image in Supplementary Fig. 5a. Dotted lines show cropped areas for each figure.

Protocol for RAISING

In our study, all enzymatic reactions in RAISING were performed using a Veriti thermal cycler (ThermoFisher Scientific).

Primer design and DNA preparation

* Use the primer design tool Primer 3[1, 2] to obtain transgene-specific primers F1, F2, and F3, with lengths of 20–27 bases and melting temperature values of approximately 68°C. These primers are typically designed to be within 500 bp of the transgene and the host genome boundary, except in the case of retroviruses or virus-based vectors that have two long terminal repeats (LTRs). The F1 primer in these exceptions should be designed outside of the LTRs to avoid amplification of the viral genome or viral vector. The F3 primer should be designed to be as close to the boundary as possible in all transgenes to obtain a longer read of the transgene-integrated host genome sequence using the Illumina HTS platform. However, we recommend designing the other F3 primer within approximately 100 bp of the boundary of the transgene and the host genome when Sanger sequencing and clonality analysis of the transgene using CLOVA will be performed instead of HTS analysis. The clonality value and Sanger sequence spectra with this F3 primer are more stable and reproducible than those with the F3 primer for HTS analysis.
* Genomic DNA should be purified using an appropriate method and treated with RNase A. We recommend checking the quality of the genomic DNA using electrophoresis before proceeding to step 1.

Step 1: Single-stranded DNA (ssDNA) synthesis

Components	Volume
Genomic DNA (200 ng/μL)	2.5 μL
10× PCR Buffer for KOD-Plus-Neo	5 μL
2 mM dNTPs	5 μL
25 mM $MgSO_4$	3 μL
10 μM F1 primer	1.5 μL
KOD-Plus-Neo (1 U/μL)	1 μL
H_2O	32 μL

Total	50 µL

Reaction condition

PCR condition	Temperature	Time	Cycles
Pre-denaturation	94°C	2 min	1
Denaturation	98°C	10 s	25
Annealing[1]	50–68°C	30 s	
Extension[2]	68°C	30 s to 2 min	

[1]The annealing step can be avoided by designing the F1 primer with a Tm value of approximately 68°C according to the manufacturer's instructions.
[2]The extension time depends on the F1 primer position.

Step 2: Column purification

・Purification of ssDNA is performed using a Monarch PCR & DNA Cleanup Kit according to the manufacturer's instructions.

・The final elution volume is approximately 8.2 µL if 9.6 µL of H_2O is used for the elution.

*The ZR-96 DNA Clean & Concentrator-5 kit (ZYMO RESEARCH) can also be used for the purification of ssDNA in the case of more than 96 specimens.

Step 3: PolyAG (TG)-tailing

・Use a single PCR tube for continuous reactions in steps 3–5.

*The polyAG-tailing can be replaced with polyTG-tailing if the transgene has a polyA sequence downstream of the specific F1 primer.

Components	Volume
ssDNA	8.2 µL
10× TdT Buffer	1.1 µL
2.5 mM $CoCl_2$	1.1 µL
10 mM dATP	0.35 µL
TdT (20 U/µL)	0.25 µL

Total	11 μL

・Incubate at 37°C for ≥ 20 min.

Components	Volume
PolyA-tailed ssDNA	11 μL
10× TdT Buffer	0.1 μL
2.5 mM $CoCl_2$	0.1 μL
10 mM dGTP	0.35 μL
H_2O	0.45 μL
Total	12 μL

・Sequentially incubate at 37°C for ≥ 15 min and then at 75°C for 10 min.

<u>Step 4: Double-stranded DNA synthesis</u>

* The NV-oligo-dA-ADP1 primer must be used if polyTG-tailed ssDNA is synthesized at step 3.

Components	Volume
PolyAG-tailed ssDNA	12 μL
5× Q5 Reaction Buffer	12 μL
10 mM dNTPs	1.2 μL
10 μM NV-oligo-dT-ADP1	3 μL
Q5 HS-High-Fidelity DNA polymerase (2 U/μL)	0.6 μL
H_2O	31.2 μL
Total	60 μL

Reaction condition

**PCR condition**	**Temperature**	**Time**	**Cycles**
Pre-denaturation	65°C	5 min	1
Annealing	64°C	10 s	
	62°C	10 s	1
	60°C	10 s	

	58°C	10 s	
	56°C	10 s	
	54°C	10 s	
	52°C	10 s	
Extension[1]	72°C	30 s to 2 min	

[1]The extension time depends on the F1 primer position.

Step 5: First PCR

* Step 5 should be performed immediately after step 4.

Components	Volume
Double-stranded DNA reaction mix	60 μL
25 μM F2 primer	1.2 μL
Total	61.2 μL

Reaction condition

PCR condition	Temperature	Time	Cycles
Pre-denaturation	98°C	30 s	1
Denaturation	98°C	5–10 s	22
Annealing[1]	50–68°C	10–30 s	
Extension[2]	72°C	30 s to 2 min	

[1]The annealing temperature depends on the Tm value of F2 primer.

[2]The extension time depends on the F2 primer position.

Step 6: Second PCR

・Make a 1/200 dilution of the first PCR products

* A consideration of PCR cycles is required based on the copy number of integrated transgenes in the genomic DNA.

Components	Volume
DNA (1/200 of first PCR reaction)	1 μL
10× PCR Buffer for KOD-Plus-Neo	5 μL
2 mM dNTPs	5 μL

25 mM $MgSO_4$	3 µL
10 µM F3 primer	1.5 µL
10 µM ADP1-HTS-R1	1.5 µL
KOD-Plus-Neo (1 U/µL)	1 µL
H_2O	32 µL
Total	50 µL

Reaction condition

PCR condition	Temperature	Time	Cycles
Pre-denaturation	94°C	2 min	1
Denaturation	98°C	10 s	26–32
Annealing[1]	50–68°C	30 s	
Extension[2]	68°C	30 s to 2 min	

[1]The annealing step can be avoided by designing the F3 primer with a Tm value of approximately 68°C according to the manufacturer's instructions.

[2]The extension time depends on the F3 primer position.

Additional step: HTS library preparation

・Re-amplify the amplicon in step 6 with two primers that contain a dual-indexed sequence (octoN) and the remainder of the Illumina adaptor sequence.

Reagents	Source	Identifier
KOD-Plus-Neo	TOYOBO	KOD-401
Monarch PCR & DNA Cleanup Kit	New England BioLabs	T1030
Terminal Transferase	New England BioLabs	M0315S
Deoxynucleotide (dNTP) Solution Set	New England BioLabs	N0446S
Q5 Hot Start High-Fidelity DNA Polymerase	New England BioLabs	M0493S

References

1. Koressaar, T. & Remm, M. Enhancements and modifications of primer design program Primer3. *Bioinformatics* **23**, 1289-1291 (2007).
2. Untergasser, A. et al. Primer3—new capabilities and interfaces. *Nucleic Acids Res* **40**, e115 (2012).
