# Supplementary material for: RAISING is a high-performance method for identifying random transgene integration sites
Source: Commun Biol. 2022 Jun 2;5:535. doi: 10.1038/s42003-022-03467-w (PMC9163355; doi:10.1038/s42003-022-03467-w)
Supplement: Supplementary file 2 — Description of Additional Supplementary Files [file 42003_2022_3467_MOESM2_ESM.pdf]

Description of Additional Supplementary Files

File name: Supplementary Data 1
Description: Primers used for Rapid Amplification of Integration Sites without Interference by Genomic DNA contamination (RAISING).

File name: Supplementary Data 2
Description: Identification of multiple transgene integration sites by Rapid Amplification of Integration Sites without Interference by Genomic DNA contamination (RAISING) with Sanger sequencing and high throughput sequencing (HTS) analysis.

File name: Supplementary Data 3
Description: Clinical profile of asymptomatic carriers (ACs), HTLV-1-associated myelopathy/tropical spastic paraparesis (HAM/TSP) patients, and adult T-cell leukemia/lymphoma (ATL) patients.

File name: Supplementary Data 4
Description: Clinical profile of progressors to adult T-cell leukemia/lymphoma (ATL) and non-progressors.
